# Induction of Innate Memory in Human Monocytes Exposed to Mixtures of Bacterial Agents and Nanoparticles

**DOI:** 10.3390/ijms232314655

**Published:** 2022-11-24

**Authors:** Giacomo Della Camera, Tinghao Liu, Wenjie Yang, Yang Li, Victor F. Puntes, Sabrina Gioria, Paola Italiani, Diana Boraschi

**Affiliations:** 1 Institute of Biochemistry and Cell Biology (IBBC), National Research Council (CNR), 80131 Napoli, Italy; 2 European Commission, Joint Research Centre (JRC), 21027 Ispra, Italy; 3 Shenzhen Institute of Advanced Technology (SIAT), Chinese Academy of Sciences (CAS), Shenzhen 518055, China; 4 China-Italy Joint Laboratory of Pharmacobiotechnology for Medical Immunomodulation (CNR, SIAT, SZN), SIAT, CAS, Shenzhen 518055, China; 5 Institut Català de Nanociència i Nanotecnologia (ICN2), Consejo Superior de Investigaciones Científicas (CSIC) and The Barcelona Institute of Science and Technology (BIST), 08036 Barcelona, Spain; 6 Vall d’Hebron Research Institute (VHIR), 08035 Barcelona, Spain; 7 Institució Catalana de Recerca I Estudis Avançats (ICREA), 08193 Barcelona, Spain; 8 Stazione Zoologica Anton Dohrn (SZN), 80121 Napoli, Italy; 9 China-Italy Joint Laboratory of Pharmacobiotechnology for Medical Immunomodulation (CNR, SIAT, SZN), IBBC, CNR, 80131 Napoli, Italy

**Keywords:** innate immunity, innate memory, nanoparticles, bacteria, LPS, monocytes, macrophages

## Abstract

We assessed whether concomitant exposure of human monocytes to bacterial agents and different engineered nanoparticles can affect the induction of protective innate memory, an immune mechanism that affords better resistance to diverse threatening challenges. Monocytes were exposed in vitro to nanoparticles of different chemical nature, shape and size either alone or admixed with LPS, and cell activation was assessed in terms of production of inflammatory (TNFα, IL-6) and anti-inflammatory cytokines (IL-10, IL-1Ra). After return to baseline conditions, cells were re-challenged with LPS and their secondary “memory” response measured. Results show that nanoparticles alone are essentially unable to generate memory, while LPS induced a tolerance memory response (less inflammatory cytokines, equal or increased anti-inflammatory cytokines). LPS-induced tolerance was not significantly affected by the presence of nanoparticles during the memory generation phase, although with substantial donor-to-donor variability. This suggests that, despite the overall lack of significant effects on LPS-induced innate memory, nanoparticles may have donor-specific effects. Thus, future nanosafety assessment and nanotherapeutic strategies will need a personalized approach in order to ensure both the safety and efficacy of nano medical compounds for individual patients.

## 1. Introduction

Innate immune cells develop an innate memory that allows them to respond better to subsequent challenges [1,2,3,4,5,6,7,8,9]. The best-known agents that induce innate memory are infectious agents and microbial compounds. In particular, bacterial lipopolysaccharide (LPS) induces a type of memory that leads to a less potent inflammatory response to a second challenge, a mechanism aiming to protect the tissues/organs from inflammation-induced damage while affording a sufficient response level [10,11,12,13,14,15,16]. The main cells that can develop innate memory are mononuclear phagocytes (monocytes and macrophages) [2,10,11,17,18], as well as Natural Killer and other Innate Lymphoid Cells [5,9,19,20,21,22], although innate memory can also be observed with non-professional immune cells such as epithelial cells [23,24,25,26,27,28]. Among mononuclear phagocytes, there is evidence that monocytes are more reactive than macrophages in memory generation [29], suggesting that the main memory cells are the effector blood monocytes (which extravasate and enter a point of inflammation in a tissue) that survive the inflammatory reaction and develop into monocyte-derived memory macrophages. Conversely, although there is evidence that naïve tissue-resident macrophages can develop memory in some circumstances [30], they may mainly act as sentinels that send alarm signals (e.g., chemokines).

The relevance of assessing the possible effects of engineered nanoparticles (NPs) on innate immunity stems from the fact that, with the explosion of nanotechnological applications, NPs are extensively produced and used in many different applications and products, which has raised concerns about their safety for human and environmental health. TiO_2_ NPs are among the most produced types of NPs worldwide [31], with applications in manufacturing and construction (e.g., car tires, concrete, sports equipment), food additives, paintings and sunscreens among others [32,33,34]. TiO_2_ NPs are generally considered safe [35,36], although their safety is currently being reconsidered [37]. CeO_2_ NPs are largely used in solar cells [38], chemical mechanical planarization [39], corrosion protection [40], fuel oxidation catalysis [41], car exhaust treatment [42] and biological sensors [43]; they are also being explored for future therapeutic applications in a range of inflammation-based diseases based on their potent anti-oxidant activity [44,45,46,47,48,49]. NPs can enter the human body by different routes, mainly inhalation and ingestion but also by skin contact (if the skin is damaged) [50,51] or, in the case of nanomedical products, by injection. Some inhaled or ingested NPs have been shown to cross biological barriers (alveolar or intestinal epithelium) and reach the blood and inner tissues [52], where they can readily interact with innate immune cells, including monocytes and macrophages [31,53]. Whether NPs can be considered immunologically safe depends on many factors, including the NP’s size, shape and composition and the concomitant presence of other bioactive agents (e.g., LPS), which can induce and modulate innate immune activation [54]. 

The direct effects of different NPs on innate immunity have been extensively studied [53,55,56], while less is known regarding their capacity to induce innate memory, although some studies have proposed that they can act as “priming” agents able to induce memory in innate immune cells [57,58,59,60,61,62,63]. In addition to a direct memory-inducing capacity, whether NPs may affect the generation of innate memory by microbial agents is an issue that still needs investigation. Even in the absence of a direct inflammatory effect, the capacity of NPs to induce or modulate innate memory is not only relevant in the general immunosafety evaluation of nanomaterials present in the human environment, but also in terms of the immunosafety of nanomedical products deliberately administered to patients.

To examine the generation of human innate memory, we have used an in vitro model that mimics the encounter between blood monocytes (representing the effector cells recruited into an inflamed tissue) and bacteria (represented by the main inflammatory molecule of gram-negative bacteria, i.e., LPS). Monocyte activation by LPS was assessed in the absence or in the presence of different types of NPs in order to examine whether NPs may have a direct effect on monocyte activation or affect LPS-induced activation. The NPs examined here include differently shaped CeO_2_ (spherical, stamp-like) and seed-like TiO_2_. The capacity of these NPs to directly induce innate memory or to affect the tolerance-type memory generated by exposure to LPS was assessed by examining cell activation in terms of alterations to the balance between inflammatory and anti-inflammatory cytokines.

The results obtained with these three types of NPs were compared with previously published results obtained in the same experimental conditions (endotoxin-free NPs pre-coated with human serum, human monocytes, LPS as a challenge) and overall show that metal NPs alone, independent of chemical composition, shape or size, have little capacity to induce innate memory and little capacity to affect the tolerance memory induced by LPS, thereby proving to be generally safe. However, some effects are detectable, which seem to be donor-dependent and exist irrespective of NP characteristics.

## 2. Results

### 2.1. Nanoparticle Characterization

**Figure 1:**
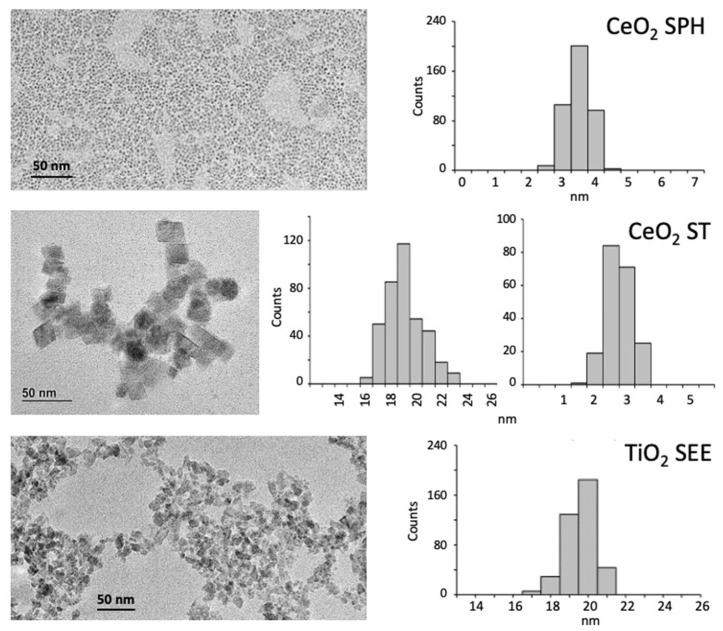
Size and morphology of the NPs used in this study. TEM images (left panels) and TEM number-weighted size distribution (right panels) of spherical CeO_2_ NPs (CeO_2_ SPH, upper panels), CeO_2_ stamp-shaped NPs (CeO_2_ STA, middle panels) and TiO_2_ seed-shaped NPs (TiO_2_ SEE, lower panels). For CeO_2_ STA, the distribution of both dimensions (side, thickness) is shown.

Three types of NPs were used in this study: CeO_2_ spherical NPs of very small size (CeO_2_ SPH, 3.5 nm), stamp-like CeO_2_ NPs that shared one dimension with the spherical NP (CeO_2_ STA, 3 × 19 nm) and seed-like TiO_2_ NPs that shared the other dimension with the stamps (TiO_2_ SEE, 19.6 nm) (Figure 1).

The NP morphological characteristics are summarized in Table 1. The NP hydrodynamic features are reported in Table 2 and show that NPs are monodispersed when suspended in their buffer (TMAOH 10 mM), except for CeO_2_ SPH that presents two additional peaks of aggregation. Suspension in culture medium leads to significant aggregation in all cases, whereas preincubation of NPs with human serum allows for a good level of dispersion and shows a modest increase in NP hydrodynamic size, attributed to the biocorona of serum proteins on the NP surface (Table 2). The number-based size distribution shows a similar profile, with serum coating affording good dispersion (Figure S1).

A single NP concentration was used for the innate memory experiments. The highest possible endotoxin-free concentration was selected as the working NP concentration. This would allow us to assess NP effects without the confounding presence of strongly inflammatory/activating endotoxin contamination, which would lead to misinterpretation of results. Endotoxin contamination of the NP preparations was measured with an optimized detection assay (Table 2), and NP concentration for the innate memory experiments was adjusted to be at or below 0.01 EU/mL of endotoxin in order to avoid endotoxin-dependent monocyte activation. In fact, human monocytes are highly sensitive to bacterial endotoxin, and even minute amounts of endotoxin (inactive in vivo or on other cells in vitro) can induce substantial activation. In addition, NP concentration was calculated in order for it to be the same for the three NPs. NP concentration was normalized to total exposed surface area, knowing that, generally, NP interactions with biological systems depend on NP surface area and characteristics. Based on these criteria, the concentration of 2.7 × 10^7^ μm^2^ of total NP surface area/mL was selected, corresponding to 0.5 μg/mL CeO_2_ SPH (corresponding to 5.9 × 10^12^ NPs/mL, with 0.011 EU/mL endotoxin), 1 μg/mL CeO_2_ STA (corresponding to 1.5 × 10^11^ NPs/mL, with 0.010 EU/mL endotoxin) and 1.6 μg/mL TiO_2_ SEE (corresponding to 1.2 × 10^11^ NPs/mL, with 0.007 EU/mL endotoxin). At these concentrations, none of the NPs showed any toxic effect on human monocytes; toxic effect was measured as LDH release after 24 h of exposure and was visually monitored by optical microscopy throughout the entirety of experimental procedures.

### 2.2. Primary Response of Human Monocytes to LPS and Nanoparticles

**Figure 2:**
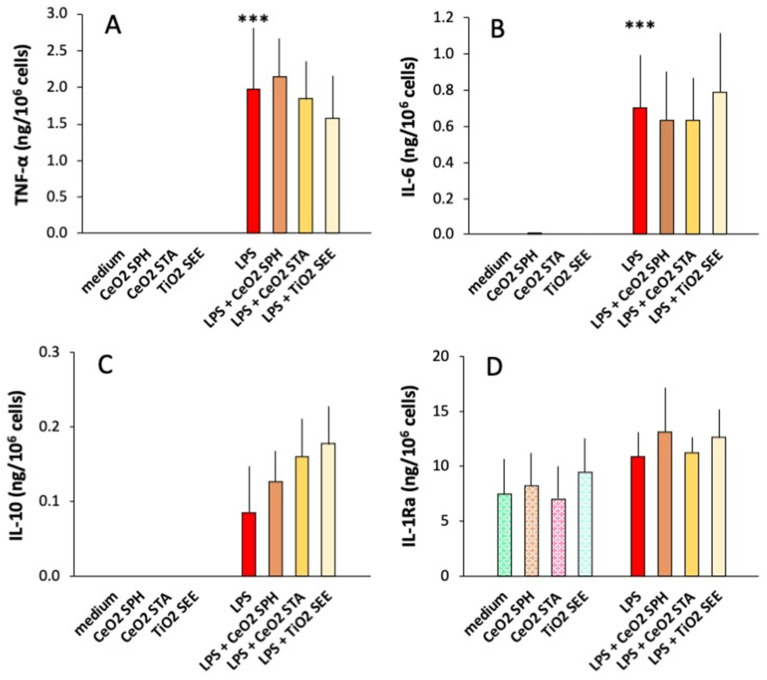
Primary innate immune response of human monocytes to LPS, NPs or their mixture. Human blood monocytes were exposed in culture to medium alone (dotted green columns) or containing 0.5 μg/mL CeO_2_ SPH (dotted orange columns), 1.0 μg/mL CeO_2_ STA (dotted pink columns), or 1.6 μg/mL TiO_2_ SEE (dotted light blue columns), or to 1 ng/mL LPS alone (red columns) or together with NPs (brown, orange and light-yellow columns). The levels of inflammatory (TNFα, IL-6; (**A**,**B**)) and anti-inflammatory cytokines (IL-10, IL-1Ra; (**C**,**D**)) were measured in the 24 h supernatants by ELISA. The columns represent the average value + SEM from three individual donors. Statistical significance: *** *p* < 0.001, medium (with or without NPs) vs. LPS (with or without NPs) for TNFα and IL-6; medium vs. LPS for IL-10 and IL-1Ra, medium vs. NPs, and LPS vs. LPS + NPs always not significant.

Results in Figure 2 show the primary response of human monocytes to NPs alone, to LPS and to LPS admixed with NPs. Production of the inflammatory cytokines TNFα and IL-6 and anti-inflammatory cytokine IL-10 was undetectable in unstimulated cells (exposed for 24 h to culture medium alone; Figure 2A–C), whereas monocytes showed a substantial constitutive production of anti-inflammatory IL-1Ra (light green dotted column in Figure 2D) as expected. Exposure to NPs did not induce production of TNFα, IL-6 and IL-10 and did not change the constitutive IL-1Ra levels. Exposure to LPS induced measurable levels of TNFα, IL-6 and IL-10, while it did not substantially increase the production of IL-1Ra. A quantitative donor-to-donor variability was observed in the response to LPS; thus, the induction of IL-10 by LPS did not reach statistical significance.

**Figure 3:**
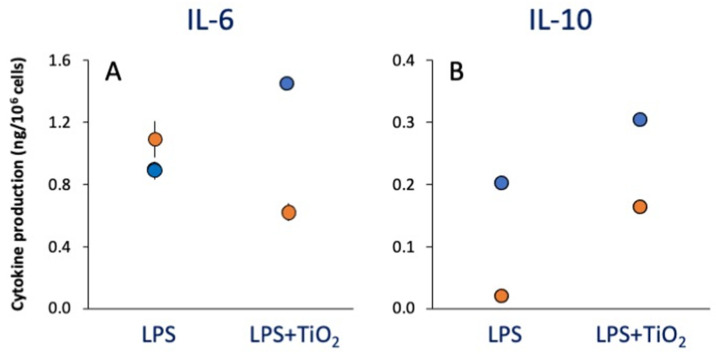
Primary innate immune response of human monocytes from individual donors to LPS, TiO_2_ SEE NPs or their mixture. Human blood monocytes from two individual donors (blue and orange dots) were exposed to LPS alone or in the presence of TiO_2_ SEE NPs, and the production of inflammatory IL-6 (**A**) and anti-inflammatory IL-10 (**B**) was measured after 24 h. Data are the average ± SD of two replicate determinations. All values of LPS + TiO_2_ SEE NPs were significantly different (*p* < 0.05) from values of LPS alone.

Co-exposure to LPS and NPs did not change the overall extent of response to LPS, although some individual effects could be observed. The results in Figure 3 show the different behavior of monocytes from two subjects in response to co-exposure to LPS and TiO_2_ SEE NPs, with cells of one subject showing a decreased production of TNFα in the presence of NPs and cells from another subject showing an increase, while the production of IL-10 was increased in both (Figure 3).

### 2.3. Memory Response of Human Monocytes to LPS and Nanoparticles

**Figure 4:**
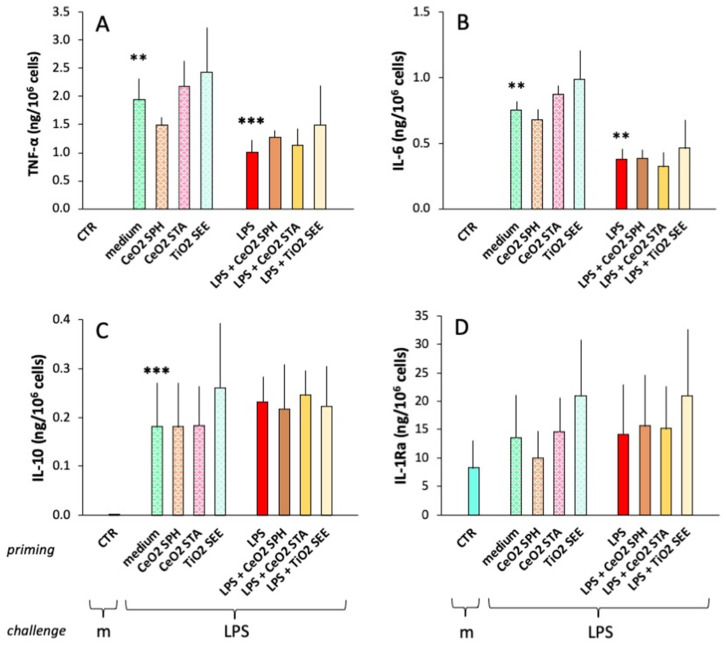
Secondary “memory” response of human monocytes primed with LPS, NPs or their mixture. After a primary exposure to NPs alone or admixed with LPS (see Figure 2; conditions indicated in the “priming” abscissa row), cells were washed and rested in culture for seven days to allow for extinction of primary activation, and then challenged for 24 h with either medium (m in the “challenge” abscissa row) or LPS (LPS in the “challenge” abscissa row). Controls (CTR; light blue columns) include cells primed with medium, buffers, NPs, LPS and LPS + NPs, which are all at baseline (as assessed upon challenge with medium), thereby confirming the complete extinction of primary activation. Inflammatory (TNFα, IL-6; (**A**,**B**)) and anti-inflammatory cytokines (IL-10, IL-1Ra; (**C**,**D**)) were measured in the 24 h supernatants by ELISA. The columns represent the average value + SEM from three individual donors. Statistical significance: ** *p* < 0.01 unchallenged controls CTR vs. medium-primed LPS-challenged (medium) for TNFα and IL-6, and for medium-primed vs. LPS-primed (LPS) for IL-6; *** *p* < 0.001, medium-primed vs. LPS-primed for TNFα, and CTR vs. medium-primed for IL-10. Other comparisons (CTR vs. medium for IL-1Ra, medium vs. LPS for IL-10 and IL-1Ra, medium vs. NPs, LPS vs. LPS + NPs) not significant.

After assessing their primary response to NPs, LPS and LPS + NPs, cells were washed and cultured with fresh medium for seven days. This period of time was selected to allow cells to undergo a complete extinction of their activation (as measured by their inability to produce cytokines above the background levels; Figure 4, CTR). In this time frame, cultured monocytes spontaneously differentiate into macrophages. Thus, we can consider them to represent the memory macrophages derived from monocytes recruited to a tissue that has experienced and resolved an infectious event. Figure 4 shows the secondary “memory” response of these macrophages to a challenge with LPS. It should be noted that the LPS concentration used for the challenge is higher than that used for the primary activation. This is meant to mimic a more severe infection so as to assess the efficacy of memory macrophages when reacting to a severe infectious event. Additionally, one should bear in mind that macrophages are generally less reactive than monocytes to microbial stimuli (to avoid excessive reaction to low-level triggering and the associated risk of unwanted tissue damage), and that they are activated towards effector functions only in response to substantial stimulation. Here, we can observe that unprimed cells (medium-primed cells, light green dotted columns) respond to the LPS challenge by producing cytokine levels that are similar to those produced by fresh monocytes in response to a 5x lower LPS concentration, which confirms the notion that macrophages are generally less responsive to challenges. Despite some donor-to-donor variability, the induction of TNFα, IL-6 and IL-10 production in response to LPS was statistically significant (Figure 4A–C), while, as expected, no increase in IL-1Ra production over the substantial constitutive production was observed (Figure 4D). When examining the memory response of NP-primed macrophages (dotted columns), we can observe essentially unchanged overall production of all cytokines relative to medium-primed cells, although again with some donor-to-donor variability. Priming with LPS (red columns) induced a typical tolerance-type memory response, characterized by decreased production of the inflammatory cytokines TNFα and IL-6 but no reduction in the production of the anti-inflammatory factors IL-10 and IL-1Ra.

**Figure 5:**
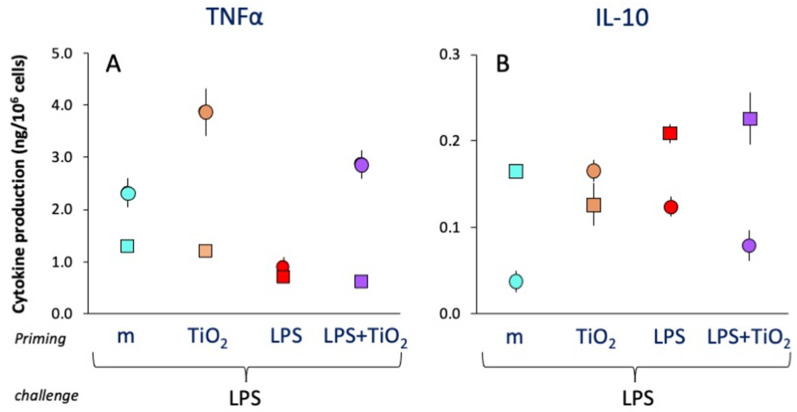
Secondary “memory” response of human monocytes from individual donors primed with LPS, TiO_2_ SEE NPs or their mixture. Human blood monocytes from two individual donors (donor 1, dots; donor 2, squares) were primed with medium alone (light blue symbols), LPS (red symbols), TiO_2_ SEE NPs (orange symbols) or their mixtures (purple symbols) and then rested and challenged with LPS, as described in Figure 4. The production of inflammatory TNFα (**A**) and anti-inflammatory IL-10 (**B**) was measured after 24 h. Data are the average ± SD of two replicate determinations. Statistical significance: TNFα: *p* < 0.05, m vs. LPS priming (light blue vs. red symbols, both donors), TiO_2_ vs. m and LPS + TiO_2_ vs. LPS priming in donor 1 (light blue and purple vs. red dots); IL-10: *p* < 0.05, m vs. TiO_2_ and LPS priming in donor 1 (light blue vs. orange and red dots). Other comparisons are not significant.

Overall, co-priming with LPS and NPs did not change the LPS priming effects (decrease in inflammatory TNFα and IL-6 production, unmodified production of anti-inflammatory IL-10 and IL-1Ra), although there were again some donor-to-donor differences. For instance, as shown in Figure 5A, cells from donor 1 (dots) seem to develop a potentiating memory to TiO_2_ NP priming in terms of the production of TNFα both alone and upon co-priming with LPS; thus, the reduction in TNFα caused by LPS tolerance (see red dot) is completely reversed in cells primed with LPS and TiO_2_ NPs (purple dot). With cells from another donor (squares), while LPS priming induced the expected tolerance-type memory (reduction of TNFα levels), TiO_2_ NPs did not induce any kind of memory, as the same level of TNFα was produced by cells primed with medium or with TiO_2_ NPs (light blue vs. orange squares) and by cells primed with either LPS or LPS and TiO_2_ NPs (red vs. purple squares). Looking at the memory effects on anti-inflammatory IL-10 production (Figure 5B), cells from one donor (dots) displayed low reactivity to the LPS challenge and showed a potentiating memory response after priming with both TiO_2_ NPs and LPS, whereas priming with the combined agents was less effective than either one alone. Cells from the other donor (medium-primed cells, light blue square) showed no effect after priming with TiO_2_ NPs, LPS or their combination. Thus, one donor (dots) seemed to develop memory in response to TiO_2_ NPs for both inflammatory and anti-inflammatory cytokines, while the combined priming was only effective on TNFα production. Conversely, cells from the other donor seemed unresponsive to TiO_2_ NPs in all conditions.

## 3. Discussion

We have compared the experimental data presented here with results obtained in previous studies performed with other NPs under the same experimental conditions, making them therefore directly comparable. The summary presented in Table 3 shows that innate memory induced by microbial agents is not generally affected by the presence of NPs, independent of their chemical nature (Au, FeOx, CeO_2_, TiO_2_), size (3–50 nm) or shape (spherical, rod, stamp, seed). The data in Table 3 also confirm the observation of a variability among donors in the NP effects on innate memory, indicated by large average variations vs. control (up to 63%) that do not reach statistical significance.

A number of considerations should be made in order to understand the relevance of these observations. First, the experimental model was designed in order to approximate real-life conditions as much as possible. Thus, human primary cells were used (excluding mouse cells and transformed cell lines), and NPs were used at concentrations in which endotoxin contamination was below functional efficacy (excluding effects not due to NPs) and were pre-exposed to human serum (to reproduce the biocorona formation that occurs when NPs enter the human body). The NP concentrations used (1.0–3.2 μg/10^6^ monocytes) are in the range expected for intravenous nanodrugs (i.e., about 10 μg/10^6^ monocytes in blood) and much higher than those foreseen for inhaled/ingested particles. 

The anti-inflammatory effects of CeO_2_ NPs described in several studies [44,45,46,47,48,49] were not observed here, as CeO_2_ NPs did not downregulate LPS-induced inflammatory activation, in line with other studies with human monocytes [64]. It is likely that the anti-inflammatory effects of CeO_2_ NPs may be evident at much higher NP concentrations than those used in studies with human primary monocytes. Thus, although the number of donors is low and some variability is observed, these data confirm all the previous data generated with other NPs and suggest that, under “physiological” dose conditions and in the presence of biological fluids, NPs are unable to directly induce an innate immune reaction or modulate a reaction induced by microbial agents, an observation that suggests their general safety.

Innate memory, the immune mechanism that allows for a more protective non-specific reaction to repeated challenges [1,2,3,4,5,6,7,8,9], was here assessed in terms of the production of two inflammatory cytokines (TNFα and IL-6) and two anti-inflammatory factors (IL-10 and IL-1Ra). Memory induced by priming with LPS is typically characterized by decreased secondary production of inflammatory factors, which aims to reduce the risk of a detrimental secondary inflammatory reaction [10,11,12,13,14,15,16]. In this study, tolerance-type innate memory was evident in the decreased production of both TNFα and IL-6, while the production of anti-inflammatory IL-10 and IL-1Ra was unaffected (Figure 4). Thus, the protective memory profile generated in response to LPS priming is represented by decreased inflammation in parallel with unchanged anti-inflammatory mechanisms. We evaluated whether co-exposure to different types of engineered NPs together with LPS interferes with the generation of this putatively protective innate memory profile and showed that NPs of different chemical composition (CeO_2_, TiO_2_) and different shape (spherical, star-like, seed-like) cannot substantially affect the ability of LPS to induce its classical memory profile. This confirms and extends previous studies with NPs of different chemical composition (gold, iron oxide), different size (12, 17, 22, 50 nm) and different shape (spherical, rod-like) (Table 3) [57,60,61,63], and underlines the general safety of NPs not only in terms of direct capacity to induce an innate inflammatory reaction, but also in terms of unwanted induction/modulation of innate memory.

From this study (Figure 3 and Figure 5) and previous results [57,60,61,63], some donor-to-donor variability in reactions to both microbial agents and NPs is evident. The fact that different individuals may develop innate memory in a different fashion, and that NPs may have effects on the microbial-induced innate memory of some individuals but not others, underlines the importance of our personal immunological history, i.e., our “immunobiography”, in determining our immune competence and capacity to react to future challenges [65]. The donor-to-donor variability observed with human subjects is not observed in inbred mice, whose macrophages seem to develop innate memory in response to TLR agonists and NPs without notable interindividual variability [59,66], or with cells from the mouse macrophage-like leukemia cell line RAW 264.7 [66]. Such observations therefore stress the degree of immunological variability in human subjects and emphasize the need for personalized assessment of NP effects on human immunity in view of future nanomedical applications and general nano-immunotoxicity assessments (at least until better knowledge is attained).

Overall, this analysis suggests that NPs may be considered as generally safe from the point of view of innate immune responses as they fail to directly induce cell activation or affect immune activation induced by bacterial molecules such as LPS. However, despite a general lack of effects, the fact NPs can modulate innate memory responses in cells from some subjects suggests caution in the medical use of NPs and the need for personalized NP safety/efficacy profiling.

## 4. Materials and Methods

### 4.1. Nanoparticle Synthesis and Characterisation

All synthesis processes were carried out using depyrogenated glassware and reagents were prepared using endotoxin-free water.

The 3.5 nm CeO_2_ spheres (CeO_2_ SPH) were synthesized by the non-isothermal precipitation procedure based on the method of Zhou et al. [67] and Chen and Chang [68], with some modifications. Briefly, 50 mL of cerium (III) nitrate solution (Ce(NO_3_)_3_·6H_2_O) 0.02 M was set at 70 °C with a stirring rate of 500 rpm and then added to 25 mL of 1M tetramethylammonium hydroxide (TMAOH), with the immediate formation of a white precipitate. Incubation was prolonged for 5 min in order to oxidize Ce(III) to Ce(IV). The solution was then rapidly transferred to a water bath, and the reaction continued at 50 °C for 16–18 h. The resulting solution was centrifuged, washed and resuspended in 50 mL 1 mM TMAOH to stabilize the formed 3.5 nm CeO_2_ NP.

CeO_2_ stamps STA (CeO_2_ STA) were synthesized in a similar way to spheres, except that TMAOH was replaced by hexamethylenetetramine (HMT), as described by García et al. [69]. Basically, Ce^3+^ ions from Ce(NO_3_)_3_ were oxidized under alkaline pH conditions to Ce^4+^ using HMT. CeO_2_ stamp-shaped NPs precipitated and were stabilized in water with HMT, which forms a double electrical layer to prevent NP aggregation.

For titanium dioxide seed-shaped NPs (TiO_2_ SEE), the synthesis procedure was based on Pottier et al. [70]. Titanium tetrachloride (TiCl_4_) was decomposed at an acidic pH (from 2 to 6), and the growth of the nanocrystals was then carried out in an oven at 70 °C. Finally, a purification step involving centrifugation and re-suspension with TMAOH was used to stabilize the NP dispersion.

NP characterization images were obtained by STEM (scanning transmission electron microscopy) using an FEI Magellan XHR microscope (FEI, Hillsboro, OR, USA) in transmission mode with an acceleration of 20 kV. Particle size was assessed using an ImageJ macro. Particle ζ-potential and hydrodynamic diameter were determined by laser doppler velocimetry and dynamic light scattering (DLS), respectively, using a Malvern Zetasizer Nano ZS instrument (Malvern Panalytical Ltd., Malvern, UK) with a light source wavelength of 632.8 nm and a fixed scattering angle of 173° (at 25 °C).

For all NP types, the main physico-chemical characteristics are reported in Figure 1 and Table 1 and Table 2. All NPs were pre-coated with human AB serum (Merck Sigma-Aldrich^®^, St. Louis, MO, USA) immediately before use in monocyte stimulation experiments. Briefly, NP suspensions were admixed 1:1 with heat-inactivated serum and incubated for 2 h at 37 °C in an orbital shaker at 500 rpm. NPs were then diluted in culture medium to the concentration required for use in the biological assays, and serum concentration was adjusted to 5%. Coating with serum significantly increased the colloidal stability of all NPs (Table 2 and Figure S1).

### 4.2. Human Monocyte Isolation

Blood was obtained from healthy donors after obtaining informed consent in accordance with the Declaration of Helsinki. The protocol was approved by the Regional Ethics Committee for Clinical Experimentation of the Tuscany Region (Ethics Committee Register n. 14,914 of 16 May 2019).

Monocytes were isolated by CD14 positive selection with magnetic microbeads (Miltenyi Biotec, Bergisch Gladbach, Germany) from peripheral blood mononuclear cells (PBMC), obtained by Ficoll-Paque gradient density separation (GE Healthcare, Bio-Sciences AB, Uppsala, Sweden). The monocyte preparations used in the experiments were > 95% viable and >95% pure (assessed by trypan blue exclusion and cytosmears). Monocytes isolated with this method were not activated, based on analysis of the expression of inflammation-related genes (*IL1B*, *TNFA*) and the release of encoded proteins, compared to monocytes within PBMC (i.e., after Ficoll-Paque separation) and whole blood (i.e., after withdrawal with anticoagulants).

Monocytes were cultured in culture medium (RPMI 1640 + Glutamax-I; GIBCO by Life Technologies, Paisley, UK) supplemented with 50 µg/mL gentamicin sulfate (GIBCO) and 5% heat-inactivated human AB serum (Merck Sigma-Aldrich^®^). Cells (5 × 10^5^) were seeded at a final volume of 1.0 mL in the wells of 24-well flat bottom plates (Corning^®^ Costar^®^; Corning Inc. Life Sciences, Oneonta, NY, USA) at 37 °C in moist air with 5% CO_2_. Monocyte stimulation was performed after resting overnight.

### 4.3. Human Monocyte Activation and Induction of Innate Memory

For assessing the primary response to stimulation, monocytes were exposed for 24 h to culture medium alone (medium/negative control) or medium containing 1 ng/mL LPS (positive control; from *E. coli* O55:B5; Merck Sigma-Aldrich^®^), serum pre-coated NPs or LPS together with NPs. 

For memory experiments, after the first exposure to stimuli for 24 h and supernatant collection, cells were washed and cultured with fresh culture medium for 7 additional days (one medium change after 4 days). During this period, the activation induced by previous stimulation subsides, and cells return to their baseline status (as determined by evaluation of inflammation-related cytokines in the supernatant). After the resting phase, the supernatant was collected, and cells were challenged for 24 h with fresh medium alone or medium containing 5 ng/mL LPS (at a 5× higher concentration than in the primary stimulation to mimic a stronger secondary challenge).

All supernatants (collected after the first stimulation, after the resting phase and after the challenge phase) were frozen at −20 °C for subsequent cytokine analysis. By visual inspection, cell viability and cell number did not substantially change in response to the different treatments.

### 4.4. Cytotoxicity Evaluation

The direct toxicity of NPs on monocytes was evaluated through the release of lactate dehydrogenase (LDH). Briefly, monocytes (1.2 × 10^5^ cells/well of 96-well plates; Corning Inc., Corning, NY, USA) were incubated for 24 h in 200 μL of culture medium alone or medium containing serum-coated NPs in triplicate. Positive control wells received 200 μL of 0.1% Triton-X 100. At the end of the incubation, release of the cytoplasmic enzyme LDH was measured in the supernatant using a colorimetric assay (LDH-Cytotoxicity Colorimetric Assay Kit; BioVision, Inc., Milpitas, CA, USA). Validation of the LDH cytotoxicity results was obtained by visual inspection of cells in culture over the entire course of the experiments. Cell density (variations in the number of cells in culture), cell refractivity, morphology and adherence (associated to live/dead cells) were assessed by phase contrast optical microscopy at 24 h (after the first stimulus), 8 days (after the resting period) and at 9 days (after the challenge).

### 4.5. Assessment of Endotoxin Contamination

Endotoxin contamination in NPs was assessed with a commercial chromogenic Limulus Amoebocyte Lysate (LAL) assay (Pyros Kinetix^®^ Flex; Associates of Cape Cod, Inc., East Falmouth, MA, USA) following a protocol adapted for use with particulate agents [71]. Preliminary controls were run to assess the possible interference of NPs in the assay readout. These encompassed direct optical reading at 405 nm (the OD of the assay readout product paranitroaniline, pNA) and interference with detection of different concentrations of synthetic pNA. NPs were used in the LAL assay at concentrations that did not interfere with the assay readout. Additional controls included assessment of the possible interference of NPs with the assay components/reagents, performed by spiking the NP samples with a known amount of LPS (0.5 EU/mL) and assessing the recovery of spiked endotoxin. A recovery rate between 80 and 120% was considered acceptable. The endotoxin contamination was therefore reliably assessed at NP dilutions that did not interfere with the 405 nm readout and in which 80–120% spiked endotoxin could be recovered. The LAL assay was run with Glucashield^®^ (Associates of Cape Cod, Inc.) using a dedicated tube reader and software (Associates of Cape Cod, Inc.) to eliminate possible false positives due to the presence of glucans. Assay sensitivity was 0.001 EU/mL.

### 4.6. Evaluation of Cytokine Production

The levels of the human inflammatory cytokines TNFα and IL-6 and anti-inflammatory factors IL-10 and IL-1Ra produced by cultured monocytes were assessed in cell supernatants by ELISA (R&D Systems, Minneapolis, MN, USA) using a Cytation 3 imaging multi-mode reader (BioTek, Winooski, VT, USA). The presence of IL-1β was not measured because, from preliminary experiments, the production of this inflammatory cytokine in cells challenged with LPS after primary activation and 7 days of resting was below detection even in controls. Each sample was tested in duplicate in ELISA.

### 4.7. Statistical Analysis

Data were analyzed using GraphPad Prism6.01 software (GraphPad Inc., La Jolla, CA, USA). For cytokine production, results are presented as ng of produced cytokine/10^6^ plated monocytes. Results are reported as the mean of values from three donors, each tested with two to six replicates. The statistical significance of differences is indicated by *p* values, which were calculated with unpaired and two-tailed Student’s *t*-tests.

## Figures and Tables

**Table 1:** Nanoparticle morphology.

Parameter	CeO_2_ SPH	CeO_2_ STA ^a^	TiO_2_ SEE
<*d*> (nm)	3.5	19.1, 2.7	19.6
σ_d_ (nm)	0.4	1.5, 0.4	0.8
σ_d_/<*d*>	9%	13%, 6%	23%
Shape	quasi-sphere	stamp	seed
Stokes diameter (nm) ^b^	2.52	38.74	20.52

The mean diameter <*d*> is reported along with the standard deviation of the diameter σ*_d_* and the coefficient of variation σ*_d_*/<*d*>. ^a^ Side and thickness ^b^ by analytical ultracentrifugation.

**Table 2:** Nanoparticle hydrodynamic features.

Parameter	CeO_2_ SPH	CeO_2_ STA	TiO_2_ SEE
*Dh*^a^ in buffer (nm) ^b^	4.2 (37.8) (142) ^c^	91.3	68.1
*Dh* in H_2_O (nm) ^d^	4.2 (28.2) (122)	106	58.8 (5560)
*Dh* in PBS 1x (nm)	4.2 (28.2) (122)	91.3	58.8 (5560)
*Dh* in medium (nm) ^e^	1480	1110	1480
*Dh* in medium plus HS (nm) ^f^	10.1 (58.8) (459)	190 (5560)	164 (5560)
ζ potential (mV) ^g^	−23.60	−40.20	−35.40
Endotoxin activity (EU/mg) ^h^	25.0	9.8	4.4

^a^ *Dh* is the intensity-based hydrodynamic size measured by DLS. ^b^ Buffer is TMAOH 10 mM for all NP types. ^c^ Between parentheses is the *Dh* of additional agglomerate peaks in DLS. ^d^ Endotoxin-free ultrapure water. ^e^ RPMI-1640 culture medium. ^f^ NPs precoated with human serum (HS) in RPMI-1640 medium. ^g^ In PBS 1x. ^h^ Endotoxin contamination of NP preparations is expressed as activity (endotoxin units, EU) per mg of NPs. All measurements were carried out in triplicate at 25 °C.

**Table 3:** Overall effects of NPs on memory cytokine production induced by microbial agents.

Priming	NPs	Memory TNFα Production		Memory IL-6 Production		Memory IL-10 Production		Memory IL-1Ra Production		N
		Variation vs. No NPs (%)	*p*	Variation vs. No NPs (%)	*p*	Variation vs. No NPs (%)	*p*	Variation vs. No NPs (%)	*p*	
LPS + NPs	Au 50 nm	−26	*ns*	nt	*nt*	nt	*nt*	nt	*nt*	4
	Au 12 nm	+35	*ns*	0	*ns*	+31	*ns*	+37	*ns*	3
	Au ROD	+63	*ns*	−4	*ns*	+15	*ns*	+2	*ns*	3
	FeOx17	−10	*ns*	+27	*ns*	+22	*ns*	+20	*ns*	3
	FeOx22	−25	*ns*	+8	*ns*	+43	*ns*	−4	*ns*	3
	CeO_2_ SPH	+1	*ns*	+2	*ns*	+1	*ns*	+33	*ns*	3
	CeO_2_ STA	−17	*ns*	−9	*ns*	+5	*ns*	+52	*ns*	3
	TiO_2_ SEE	−19	*ns*	+25	*ns*	−8	*ns*	+24	*ns*	3
MDP + NPs	Au 50 nm	−39	*<0.05*	nt	*nt*	nt	*nt*	nt	*nt*	4
β-glucan + NPs	Au 50 nm	+30	*ns*	nt	*nt*	nt	*nt*	nt	*nt*	4
*H. pylori* + NPs	Au 50 nm	0	*ns*	0	*ns*	−30	*<0.05*	+3	*ns*	12
*S. aureus* + NPs	Au 50 nm	−4	*ns*	nt	*nt*	nt	*nt*	nt	*nt*	8
*C. albicans* + NPs	Au 50 nm	+9	*ns*	+8	*ns*	+12	*ns*	+4	*ns*	4

The table reports a summary of NP effects of innate memory (assessed as production of inflammatory and anti-inflammatory cytokines) induced in human monocytes by microbial stimuli. LPS priming data are taken from Swartzwelter et al. [61] for spherical 50 nm AuNPs, from Della Camera et al. [63] for spherical 12 nm AuNPs, rod-shaped AuNPs (20 × 13 nm) and spherical FeOx NPs of 17 and 22 nm; data for CeO_2_ and TiO_2_ NPs are from this study. All other data, with spherical 50 nm AuNPs, are from Swartzwelter et al. [61]. All NPs were used at the maximal endotoxin-free concentration, i.e., 20 μg/mL for 50 nm AuNPs, 5.7 μg/mL for 12 nm AuNPs, 1.4 μg/mL for ROD AuNPs, 2.0 μg/mL for FeOx17 NPs, 2.7 μg/mL for FeOx22 NPs, 0.5 μg/mL for CeO_2_ SPH NPs, 1.0 μg/mL for CeO_2_ STA NPs and 1.6 μg/mL for TiO_2_ SEE NPs. After priming, cells were rested in culture for 6–7 days and then challenged with 5–10 ng/mL of LPS. Memory cytokine production was measured after 24 h. NP-dependent variations in memory cytokine production are reported as the percentage of response in cells primed in the absence of NPs and are the average of data from 3–12 donors (donor number N reported in the right column; SD not reported). Variations above (+) and below (−) the values in the absence of NP priming are never statistically significant (*ns* in the *p* columns). A limited but significant decrease was only observed for 50 nm AuNPs in TNFα production in cells primed with the gram-positive bacterial molecule muramyl dipeptide (MDP), suggesting tolerance-type memory (decreased inflammation), and in IL-10 production in cells primed with the gram-negative bacteria *H. pylori*, suggesting a more inflammatory secondary reaction. nt, not tested.

## Data Availability

Data supporting reported results can be found in the supplementary material and are available from the authors upon request.

## Supplementary Materials

The supporting information can be downloaded at: https://www.mdpi.com/article/10.3390/ijms232314655/s1.
