# Supplementary material for: Induction of Innate Memory in Human Monocytes Exposed to Mixtures of Bacterial Agents and Nanoparticles
Source: Int J Mol Sci. 2022 Nov 24;23(23):14655. doi: 10.3390/ijms232314655 (PMC9738562; doi:10.3390/ijms232314655)
Supplement: Supplementary file 1 [file ijms-23-14655-s001.zip › ijms-1972575-supplementary.pdf]

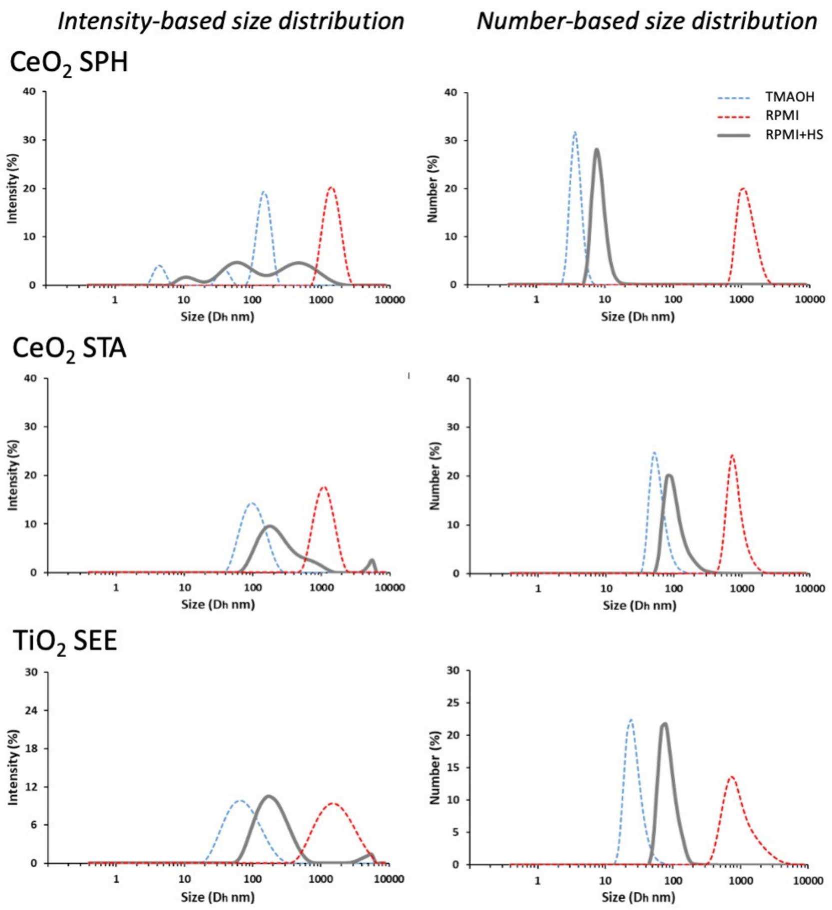

Supplementary Figure S1. **Particle size distribution of NPs in different media**. The averaged (n=3) intensity-based (left panels) and number-based (right panels) particle size distribution is shown, measured by batch-mode DLS. The hydrodynamic diameter (*Dh*, in nm) was assessed of particles in their original buffer solution TMAOH 10 mM (TMAOH; dashed blue line), suspended in RPMI-1640 culture medium (RPMI; dashed red line) and in RPMI after pre-coating with human serum (RPMI+HS; solid line).
